# The effect of dental trauma management resources on dental practitioners’ self-reported confidence and knowledge in the United Arab Emirates

**DOI:** 10.3389/fdmed.2026.1883091

**Published:** 2026-07-16

**Authors:** Ahmed Fayaz, Iyad Hussein, Manal Al Halabi, Anas Salami, Hani Nazzal, Mawlood Kowash

**Affiliations:** 1 Hamdan Bin Mohammed College of Dental Medicine, Mohammed Bin Rashid University of Medicine and Health Sciences, Dubai Health, Dubai, United Arab Emirates; 2 Hamad Medical Corporation, Doha, Qatar

**Keywords:** dental trauma guide, dentists, IADT guidelines, knowledge, traumatic dental injuries, United Arab Emirates

## Abstract

**Objectives:**

To assess dental practitioners’ knowledge, confidence, and use of evidence-based resources, specifically the International Association of Dental Traumatology (IADT) guidelines and the Dental Trauma Guide (DTG), in the management of traumatic dental injuries (TDIs) in the United Arab Emirates (UAE).

**Methods:**

A cross-sectional survey was conducted among licensed general dental practitioners, pediatric dentists, and endodontists in the UAE. A validated, self-administered questionnaire was distributed via multiple recruitment channels. The instrument collected demographic information, self-reported confidence levels, awareness, and use of trauma-management resources, as well as responses to two clinical scenarios. Data was analyzed using descriptive statistics and the Wilcoxon Signed-Rank Test; *p* < 0.05 was considered significant.

**Results:**

A total of 108 dentists participated (response rate of 54%). Overall awareness of the IADT guidelines and DTG was moderate (50.5% and 58.3%, respectively). Pediatric dentists demonstrated significantly greater confidence in managing both simple and complex TDIs than general practitioners and endodontists (*p* < 0.01). Dentists with ≤5 years of experience reported significantly lower confidence in managing complex injuries (*p* = 0.005). Limited clinical exposure and lack of formal training were the most frequently reported barriers to confidence. Case scenario responses revealed notable gaps in evidence-based decision-making, particularly in the management of complex crown fractures and intruded primary incisors.

**Conclusion:**

Dentists in the UAE exhibit variable knowledge and confidence in TDI management, with inconsistent use of evidence-based guidelines. Specialty, experience, and exposure to trauma cases strongly influenced confidence and decision-making. These findings underscore the need for enhanced undergraduate training, ongoing education, and broader dissemination of the IADT guidelines and DTG to enhance the quality of dental trauma care in the UAE.

## 1 Introduction

A traumatic dental injury (TDI) represents a major public oral health concern due to its high incidence, early onset, long-term treatment needs, and associated costs (1). TDIs constitute a substantial proportion of bodily injuries (2), with 85% of affected individuals sustaining trauma to the oral region (3). Although the oral cavity represents only 1% of total body surface area, oral injuries account for 5% of all bodily trauma and up to 17% among preschool-aged children (4). Globally, TDIs affect approximately 1%–3% of the population, with children and adolescents being disproportionately impacted (5). It is estimated that more than one billion individuals have experienced a TDI (2).

Following dental caries, TDIs are considered the second-most-prevalent oral health condition and would rank among the world's most common acute and chronic disorders if formally listed (2). Glendor notes that one-third of children experience TDIs in their primary dentition, while one-fourth of school-aged children and one-third of adults are affected in the permanent dentition (1). By age 14, nearly every child is estimated to have sustained some form of dental trauma (6). The global prevalence of TDIs is reported as 22.7% in primary teeth and 15.2% in permanent teeth (2).

Prompt diagnosis and appropriate treatment planning are essential for achieving favorable outcomes (7). Delayed or inadequate management can result in complications such as pulp necrosis and increased long-term treatment costs (8–11). Consequently, it is crucial that dental practitioners possess strong, evidence-based knowledge to deliver optimal care (12).

Despite this need, research consistently shows that general dental practitioners (GDPs) demonstrate only low to moderate knowledge of TDI management (13–18). A study in Brazil found that dentists had only moderate familiarity with the International Association of Dental Traumatology (IADT) guidelines (13), while a UAE-based study reported poor knowledge among both GDPs and pediatric dentists (PDs) (16). Similar deficiencies have been observed in several other countries (14, 15, 17, 18). Additional work has explored dental practitioners' knowledge, confidence, and perceptions regarding TDI management (19–23). Given the complexity and variability of traumatic dental injuries, management often requires input from specialized dentists, particularly pediatric dentists and endodontists, who are more likely to encounter and manage such cases in clinical practice. Their advanced training and experience play a critical role in accurate diagnosis, evidence-based treatment planning, and long-term follow-up, making their level of knowledge and confidence in TDI management especially important.

The Dental Trauma Guide (DTG), launched in 2008 by the University Hospital of Copenhagen, is a web-based platform that provides updated clinical guidance and multimedia resources for managing TDIs (24). A recent study evaluating the impact of the IADT guidelines and DTG among GDPs in Gulf Cooperation Council (GCC) countries (excluding the UAE) found that DTG use significantly improved practitioners' confidence while also highlighting persistent knowledge gaps (12).

To date, no study has assessed the awareness or application of the IADT guidelines and DTG among GDPs, PDs, and endodontists (EDs) in the United Arab Emirates. This study seeks to address this gap.

## 2 Material and methods

This cross-sectional study used a structured, self-administered questionnaire and adhered to the E (Strengthening the Reporting of Observational Studies in Epidemiology) checklist for cross-sectional research (25). Ethical approval was obtained from the Institutional Review Board of Mohammed Bin Rashid University of Medicine and Health Sciences (MBRU IRB-2022-180).

Licensed GDPs, PDs, and EDs registered with the UAE health authorities were invited to participate in this study. Recruitment was conducted through multiple outreach channels, including social media platforms, national dental conferences, and professional society databases, to ensure broad representation across the dental community.

The required sample size was estimated under the conservative assumption that 50% of dentists belonged to each practice category, given the absence of UAE-specific distribution data. This assumption maximizes sample size and statistical power. Based on a finite population of approximately 6,800 dental practitioners, a 10% precision margin, and a 95% confidence level, a minimum sample size of 95 was calculated. This approach is consistent with the methodology used in a recent comparable survey (26). If 50% of a sample of 95 respondents belongs to a specific category, the true population proportion lies between 40% and 60% with 95% confidence.

All participants viewed an online informed consent form that explained the study's purpose, ensured their anonymity, and emphasized the voluntary nature of their participation. Permission was obtained to use a validated questionnaire from a previously published study (12) in *Dental Traumatology*. The questionnaire had been piloted among dentists in the Gulf Cooperation Council (GCC) region to establish face and content validity, as reported by Elwerfelli et al. (12).

The questionnaire, administered via Microsoft Forms®, included closed-ended questions divided into two main sections. The first collected demographic and professional information, including gender, age, practice setting, years of experience, attendance at continuing education courses on dental trauma, and self-reported confidence in managing TDIs in both primary and permanent dentitions. Confidence levels were rated on a 5-point Likert scale (1 = not confident at all to 5 = very confident).

This section also assessed participants' awareness of the 2020 IADT guideline update and their use of the Dental Trauma Guide (DTG).

The second section consisted of two clinical case scenarios designed to evaluate participants' knowledge of evidence-based TDI management in primary and permanent dentitions, in accordance with IADT recommendations. Structure and detailed items of the questionnaire are presented in Supplementary Table S1.

Participation was entirely voluntary. No identifying information was collected, and no follow-up reminders were issued. Data were exported to SPSS for Windows, version 25.0 (SPSS Inc., Chicago, IL), for statistical analysis. Descriptive statistics were used to summarize demographic characteristics and survey responses. Differences in self-reported confidence between simple and complex cases were examined using the Wilcoxon Signed-Rank Test. Statistical significance was set at *p* < 0.05.

## Results

A total of 108 licensed dentists completed the questionnaire, resulting in a response rate of 54%. The survey demonstrated excellent internal consistency, as indicated by a Cronbach's alpha coefficient of 0.92, suggesting high reliability among the questionnaire items.

### Demographic characteristics

Table 1 presents the demographic profile of respondents. Most participants were female (79.6%) and based in Dubai (66.6%). The largest age group was 31–40 years (38.9%). GDPs comprised 54.6% of the sample, followed by PDs (27.8%) and EDs (17.6%). Nearly half of the respondents (45.4%) reported practicing 29–35 h per week, and 34.3% had five or fewer years of experience.

**Table 1:** Demographic and professional profiles of respondents.

Variable	*N* (%)*
Gender	
Male	22 (20.40)
Female	86 (79.60)
Age	
20–30	35 (32.4)
31–40	42 (38.9)
41–50	24 (22.2)
>50	7 (6.5)
Speciality status	
Paediatric dentist	30 (27.8)
GDP	59 (54.6)
Endodontist	19 (17.6)
Practice location	
Abu Dhabi	8 (7.4)
Dubai	72 (66.6)
Al Sharjah	9 (8.3)
Ajman	9 (8.3)
Ras Al Khaimah	1 (0.9)
Al Fujairah	6 (5.6)
Umm Al Quwain	3 (2.8)
Type of practice	
Government hospital	31 (28.7)
Private practice	47 (43.5)
University teaching hospital	29 (26.9)
Others	1 (0.9)
Number of hours in practice per week	
≤7	10 (9.3)
8–14	16 (14.8)
15–21	8 (7.4)
22–28	25 (23.1)
29–35	49 (45.4)
Years of experience in years	
≤5	37 (34.3)
6–10	31 (28.7)
11–20	27 (25)
≥21	13 (12)

* Percentages between parentheses are rounded and included for comparison.

### Exposure to dental trauma and training background

As shown in Table 2, almost two-thirds (63.9%) reported seeing 0–4 dental trauma cases over a 3-month period. A similar proportion (63.9%) had not received postgraduate training in dental trauma, and over half (53.7%) had not attended continuing education related to TDIs. Most dentists (72.2%) had access to a pediatric dentist for clinical support in trauma cases.

**Table 2:** Dental trauma profiles among respondents.

Variable/question	*N* (%)
On average, the number of dental traumas seen in a three-month period	
0–4	69 (63.9)
5–12	23 (21.3)
13–24	12 (11.1)
>24	4 (3.7)
Dental trauma management training after graduation	
No	69 (63.9)
Yes	39 (36.1)
Attending the dental trauma management continuous educational course	
No	58 (53.7)
Yes	50 (46.3)
Having a pediatric dental specialist's support at the workplace	
No	30 (27.8)
Yes	78 (72.2)
How often do you use the online dental trauma guide in treating trauma cases?	
Rarely (1%–24% of cases)	31 (28.7)
Sometimes (25%–49% of cases)	14 (13)
Often (50%–74% of cases)	32 (29.6)
Almost always (75%–100% of cases)	31 (28.7)
Awareness about the update of the international dental traumatology guidelines 2020	
Aware and have not read it yet	22 (21.8)
Aware and read it	44 (43.6)
Unaware of the recent update	35 (34.4)
Awareness about the online dental trauma guide developed by the International Association of Dental Traumatology	
No	36 (35.6)
Yes, aware of it and use it	51 (50.5)

### Awareness and use of the dental trauma guide and IADT guidelines

Awareness and utilization of the Dental Trauma Guide (DTG) varied. Overall, 58.3% were familiar with DTG, and half (50.5%) reported using it in more than half of their trauma cases. However, 34.4% were unaware of the 2020 IADT guideline update.

### Confidence in managing traumatic dental injuries

Respondents reported high confidence in managing uncomplicated primary crown fractures involving enamel and dentine (mean 4.24; 85% confident). Confidence also remained high for complicated primary crown fractures (mean 4.06; 81% confident). Confidence scores for other primary tooth injuries, including crown-root fractures, root fractures, concussion, subluxation, lateral luxation, extrusion, intrusion, and avulsion, ranged from neutral to confident (means 3.65–3.99).

**Figure 1:**
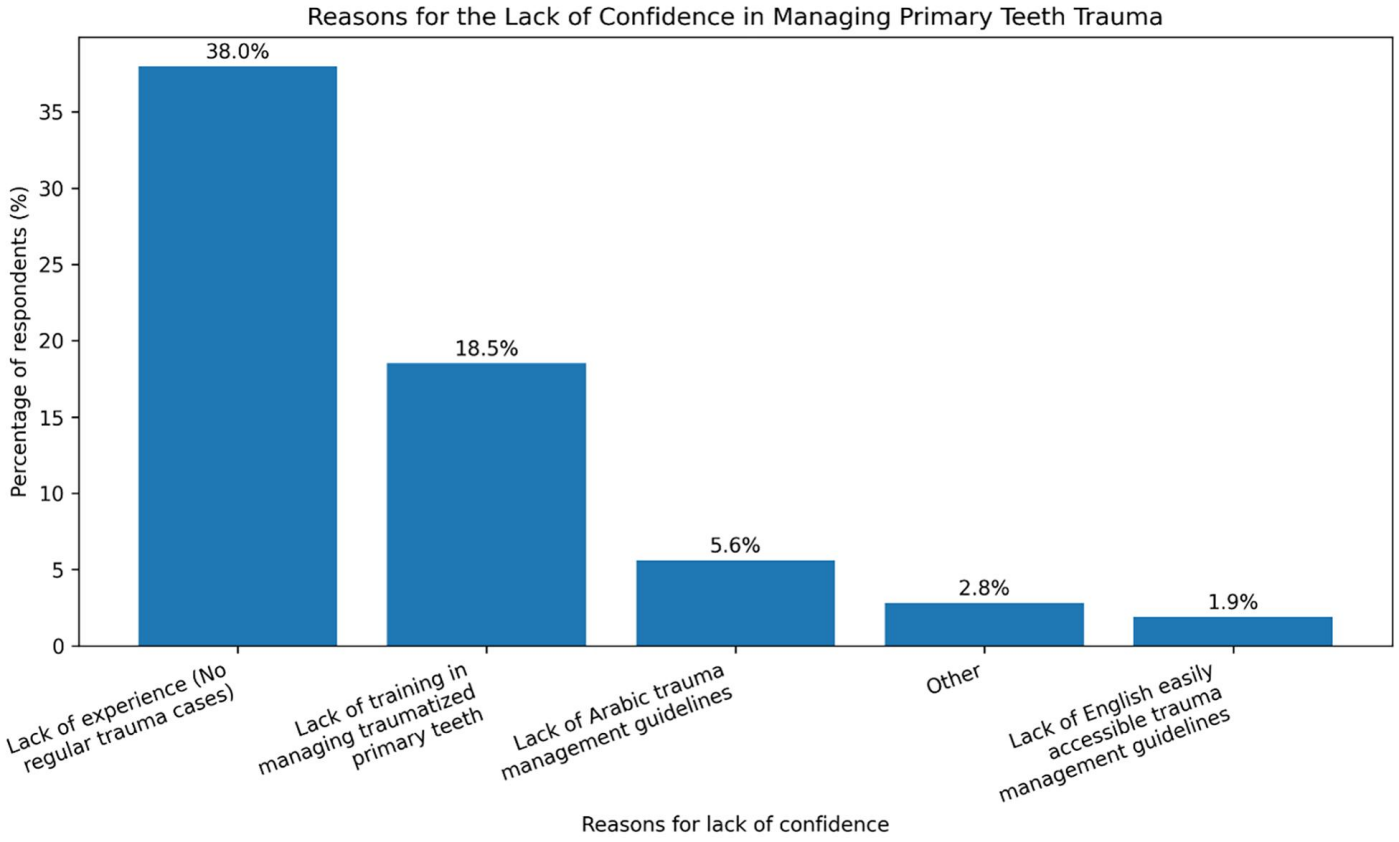
Reasons for lack of confidence in managing primary tooth trauma.

Complex cases, particularly crown-root fractures, root fractures, and alveolar bone fractures, elicited lower confidence (means 3.13–3.81). Figure 1 summarizes factors contributing to reduced confidence, with limited clinical exposure (38.0%) and lack of formal training (18.5%) being the most frequently cited reasons.

In the permanent dentition, the highest confidence was reported for performing partial (Cvek) pulpotomy (mean 3.97; 79% confident). Neutral confidence levels were observed for surgical repositioning of intruded teeth (mean 3.06; 61%) and decoronation for infra-occluded teeth (mean 2.99; 60%). Lower confidence was reported for MTA apical plug placement (mean 2.89; 58%) and regenerative endodontic procedures (mean 2.98; 60%). Splinting of traumatized teeth had comparatively higher confidence (mean 3.77; 75%).

**Figure 2:**
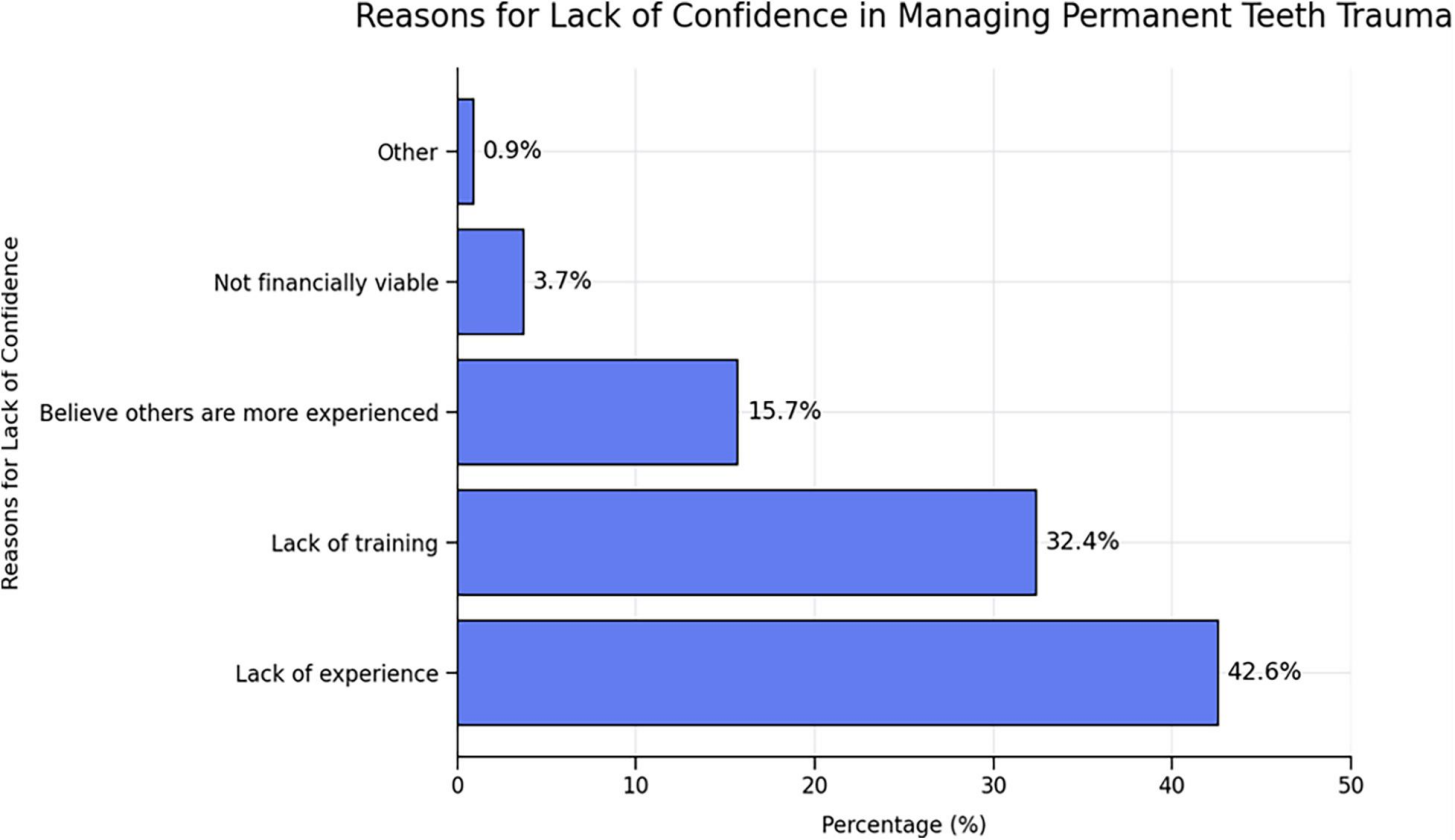
Reasons for lack of confidence in managing permanent tooth trauma.

As shown in Figure 2, reduced confidence in managing permanent TDIs was attributed primarily to limited clinical experience (42.6%) and insufficient training (32.4%).

### Influence of demographic factors on confidence

Table 3 summarizes associations between demographic variables and self-reported confidence. PDs consistently reported the highest confidence in both simple (*p* = 0.02) and complex injuries (*p* = 0.0035). Dentists with ≤5 years of experience showed significantly lower confidence in managing complex injuries (*p* = 0.005). Younger practitioners (20–30 years) also demonstrated lower confidence compared to older age groups (*p* = 0.0075). Practice setting influenced confidence, with dentists in private clinics reporting lower confidence in managing complex cases compared to those in government hospitals (*p* = 0.04).

**Table 3:** Confidence and demographics in managing simple and complex TDIs.

Variable	Simple injuries	Complex injuries	*p*-value
	Median (IQR)	Median (IQR)	
Gender			
Male	4 (1)	4 (1)	0.4141
Female	4 (1.5)	3.42 (1.21)	0.0902
***P-value***	***0.079***	***0.017***	
Age			
20–30	3.63 (1.75)	3.14 (1.04)	0.0075
31–40	4.25 (1.19)	4 (1.14)	0.0445
41–50	4 (1.5)	4 (1.5)	0.4712
>50	5 (1)	4.14 (1.71)	0.3811
***P-value***	***0.031***	***0.002***	
Place of practice			
Government hospital	4 (1.5)	4 (1.43)	0.0727
Private practice	4 (1.5)	3.86 (1.39)	0.0407
University teaching hospital	3.88 (1.75)	3.36 (1.25)	0.0624
***P-value***	***0.019***	***0.013***	
Number of hours in practice per week			
≤7	4 (2)	3.29 (1.14)	0.4529
8–14	4 (1.81)	3.57 (1)	0.2215
15–21	3.5 (2.13)	3.29 (2.07)	0.2253
22–28	4 (1.69)	3.5 (1.43)	0.4722
29–35	4.38 (1.19)	4 (1.29)	0.0033
***P-value***	*0.516*	*0.147*	
Position of the respondent			
Paediatric dentist	4.88 (1)	4 (1.11)	0.0200
GDP	4 (1.25)	3.29 (1)	0.0035
Endodontist	4.25 (1.75)	4 (2.14)	0.7264
Others	3.5 (1.81)	3.12 (1.18)	0.4052
***P-value***	***0.021***	***0.002***	
Years of experience in years			
≤5	3.75 (1.75)	3.29 (1.07)	0.0051
6–10	4.13 (1.31)	4 (1.32)	0.1929
11–20	4 (1.25)	4 (1.43)	0.1662
≥21	4.25 (1.38)	4 (1.79)	0.3997
***p-value***	*0.527*	***0.009***	

Bold values indicate statistically significant differences (*p* < 0.05).

### Trauma guideline and resources utilization

**Figure 3:**
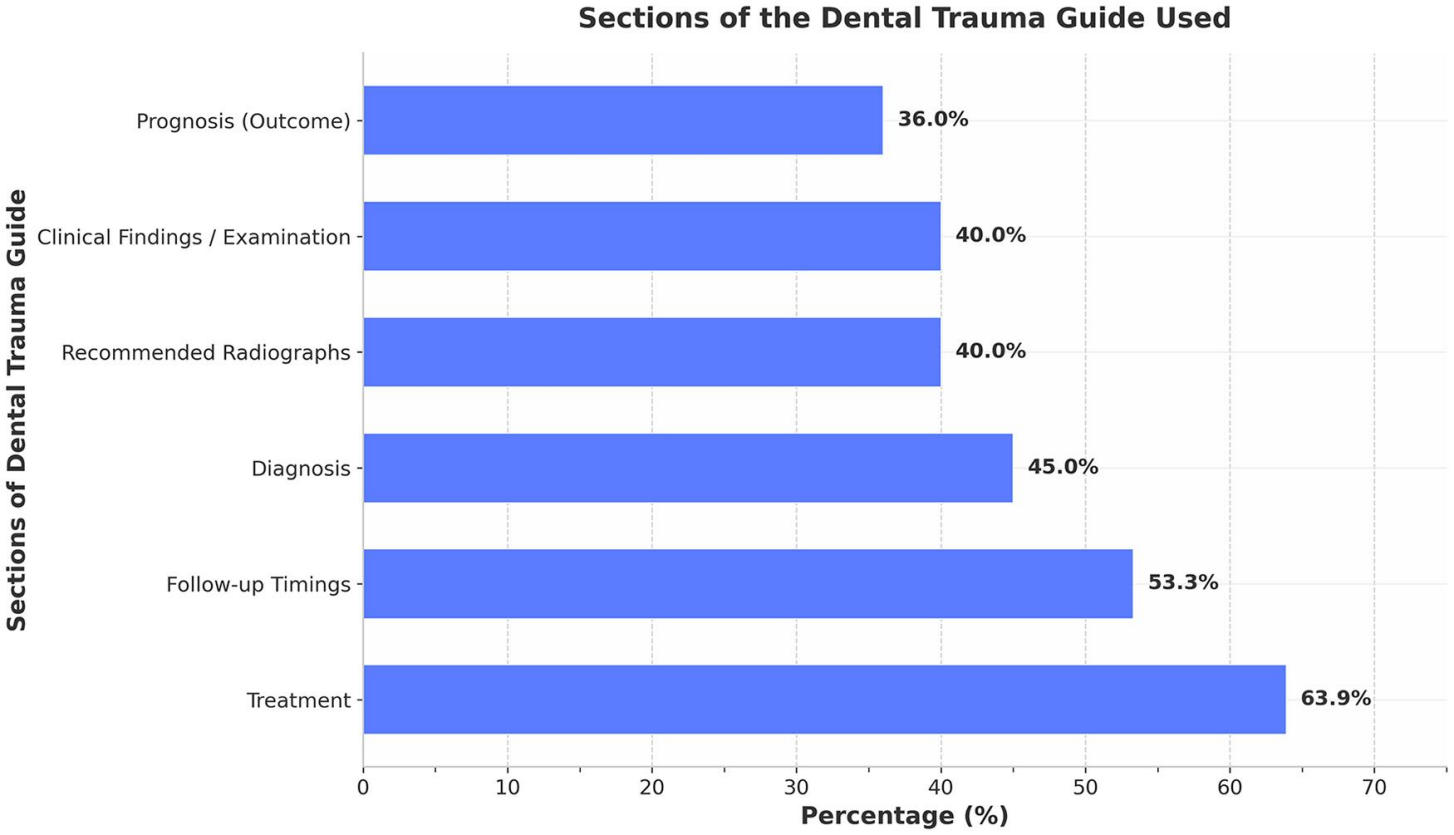
Commonly used sections of the dental trauma guide by the participants.

Figure 3 illustrates how practitioners incorporated IADT guidelines into clinical practice, with treatment recommendations (63.9%) and follow-up protocols (53.3%) being the most referenced components. Dentists who used the DTG reported significantly higher confidence in managing both simple and complex injuries compared with non-users (median 4.0 vs. 3.3, *p* < 0.05).

### Barriers to DTG utilization

**Figure 4:**
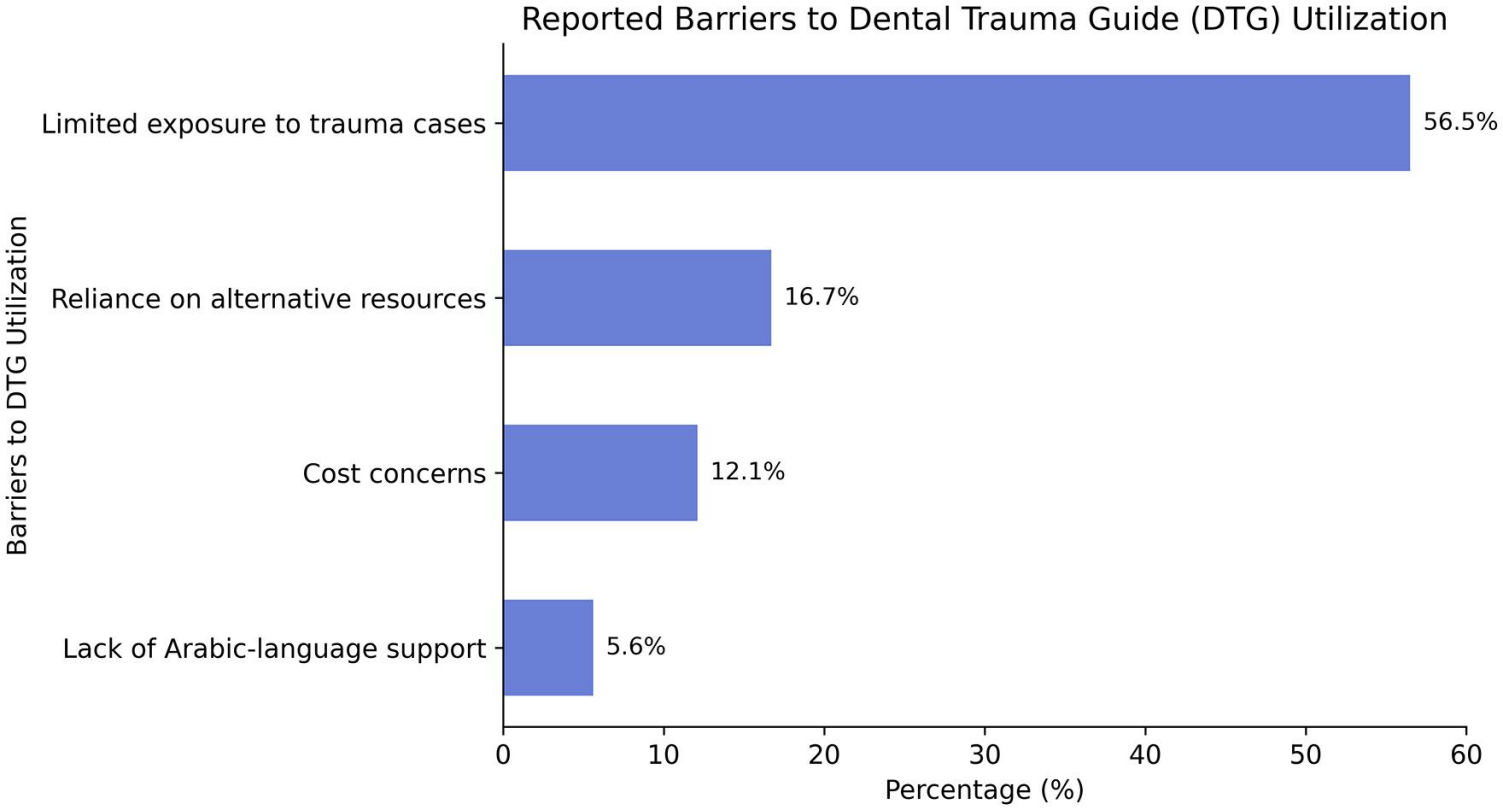
Reported barriers to dental trauma guide (DTG) utilization.

The most common barrier reported by the participants for not using DTG (Figure 4) was limited exposure to trauma cases (56.5%). Additional barriers included reliance on alternative resources (16.7%), cost concerns (12.1%), and a lack of Arabic-language support (5.6%).

### Case scenario responses

In the scenario involving a 7-year-old child with a complicated crown fracture (Table 4), 65% selected partial pulpotomy (Cvek pulpotomy), in accordance with current recommendations. Pulp extirpation (19.4%) and direct pulp capping (13.9%) were also selected. No significant association was found between specialty and response accuracy (*p* = 0.16).

**Table 4:** Case scenario responses and associated factors among participants.

Case Scenario	Clinical Option/Response	Percentage (%)	Correct According to Guidelines
A 7-year-old child with a complicated crown fracture (permanent tooth)	Partial pulpotomy (Cvek pulpotomy)	65.0	Yes
	Pulp extirpation	19.4	No
	Direct pulp capping	13.9	No
Intruded primary incisor	Spontaneous re-eruption	62.3	Yes
	Extraction (palatal intrusion)	27.4	No
	Extraction regardless of direction	10.4	No

Regarding the management of an intruded primary incisor (Table 4), 62.3% correctly opted for spontaneous re-eruption as recommended by recent guidelines, while 27.4% recommended extraction of a palatally intruded tooth, and 10.4% suggested extraction regardless of displacement direction.

Specialty was significantly associated with incorrect responses (*p* = 0.004), with GDPs accounting for the majority of inaccurate responses. Practice location also influenced accuracy, with respondents in Dubai more likely to answer incorrectly (*p* = 0.049).

### Interest in further education

**Figure 5:**
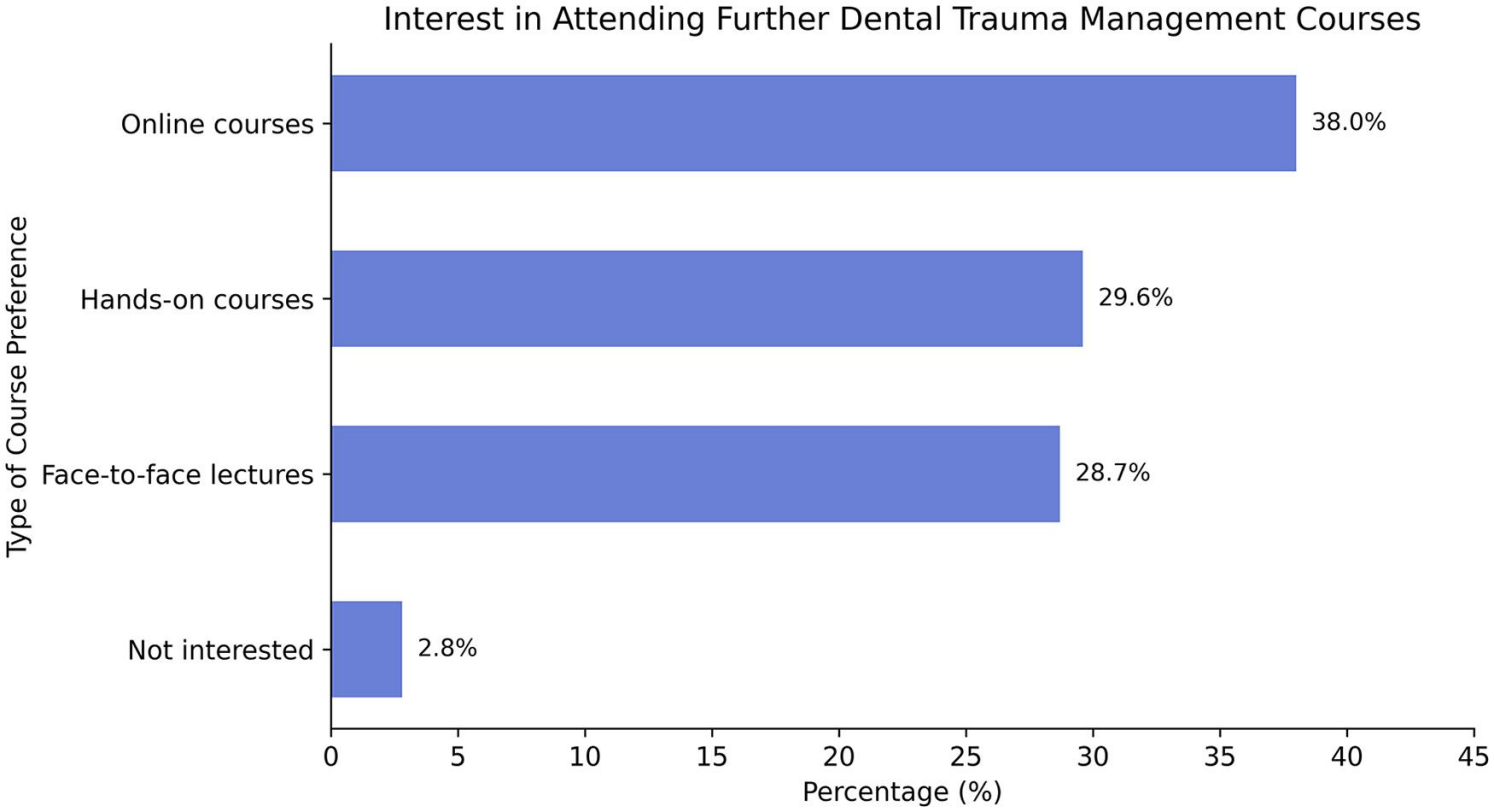
Participants’ interest in further dental trauma management courses.

Figure 5 shows that most respondents expressed interest in further education on dental trauma management. Online courses were the preferred option (38%), followed by hands-on workshops (29.6%) and face-to-face lectures (28.7%). Only 2.8% reported no interest in additional training.

## Discussion

This study highlights substantial gaps in knowledge, confidence, and the use of evidence-based resources for managing traumatic dental injuries (TDIs) among dental practitioners in the UAE. Despite the global burden of TDIs, particularly in developing regions, relatively little research has examined how access to trauma-management tools influences practitioners' perceived competence (12). Consistent with the findings of Alyasi et al. (16), the present study identified no notable improvement in TDI-related knowledge among UAE dentists. However, this is the first study in the UAE to assess awareness and use of both the updated International Association of Dental Traumatology (IADT) guidelines and the Dental Trauma Guide (DTG) among GDPs, PDs, and EDs.

The lack of improvement in TDI-related knowledge compared with 2018 may reflect persistent gaps in undergraduate training, limited exposure to trauma cases, the introduction of the 2020 IADT new guidance, the diverse background of the dental practitioners in Dubai, and inconsistent adoption of evidence-based resources such as IADT guidelines. These findings suggest that passive availability of guidelines alone is insufficient and highlight the need for structured, targeted educational interventions and improved knowledge translation strategies.

The IADT guidelines are widely regarded as the gold standard for evidence-based dental trauma care (24). Nonetheless, integrating these guidelines into clinical practice remains inconsistent. Like previous studies, our results revealed inadequate knowledge among general practitioners (12, 16, 26). By including a broader range of specialties and using a validated instrument developed by Elwerfelli et al. (12), this study builds on existing literature and provides a more comprehensive understanding of knowledge, confidence, and clinical behaviors across different practitioner groups.

The 54% response rate reflects high engagement for an online cross-sectional survey, and the high internal consistency (Cronbach's Alpha = 0.92) reinforces the instrument's reliability. However, non-response bias remains a possibility, as individuals may have declined participation due to hesitation, time constraints, or difficulties interpreting survey items, as noted by Abramson (27).

Female dentists comprised most respondents. While Smith (28) reported that women are generally more likely to participate in surveys, this may also reflect the demographic profile of the sample accessed. Similar gender-imbalanced patterns have been observed in previous studies (29, 30). Interestingly, male dentists in the present study reported greater confidence in managing TDIs, which aligns with the findings of Karaharju-Suvanto et al. (31). However, this must be interpreted cautiously due to the uneven gender distribution. Other studies have reported contrasting findings: Al-Haj et al. (14) found that female dentists demonstrated superior TDI knowledge, while Jadav and Abbott (26) observed no significant gender-based differences.

Age appeared to influence confidence, with younger dentists (20–30 years) reporting lower confidence, particularly in complex cases. Nevertheless, Jadav and Abbott (26) found no correlation between age and knowledge, suggesting that age alone may not be a reliable predictor of competence. The relatively high proportion of pediatric dentists (27.8%) in our sample likely contributed to stronger performance in TDI-related knowledge, given their routine involvement in managing dental trauma among children.

Years of professional experience also influenced confidence. Dentists with ≤5 years of experience demonstrated significantly lower confidence in managing complex injuries, a finding consistent with Al-Haj et al. (14), who reported a positive association between experience and clinical confidence. These findings underscore that clinical confidence is shaped by multiple factors, including age, clinical exposure, specialty, and practice environment.

Specialty was strongly associated with knowledge levels. PDs consistently showed higher proficiency in emergency TDI management, corroborating previous studies that identified knowledge gaps among GDPs (12, 16, 17, 32). Interdisciplinary management is particularly important in complex traumatic dental injuries requiring coordinated care among different dental specialties (33).

Practice setting also influenced confidence, with private-sector dentists reporting significantly lower confidence in complex cases. This may reflect limited access to structured training or a greater focus on procedures with higher financial returns, such as orthodontics or aesthetic treatments, which involve fewer complications and less follow-up.

A professional role further differentiated confidence in managing simple and complex TDIs. PDs demonstrated the highest median confidence scores, followed by endodontists, whereas GDPs reported the lowest confidence levels. This pattern aligns with findings from Al-Haj et al. (14), though it contradicts results from Jadav and Abbott (26), who observed no significant differences between specialists and GDPs. These discrepancies may reflect variations in training opportunities and access to continuing education across regions.

Lower exposure to complex trauma cases likely contributed to reduced confidence among respondents. Consistent with the findings of Elwerfelli et al. (12), this study found that limited clinical experience remains a primary barrier to confidence in managing TDIs. The strong association between exposure and confidence reinforces the importance of hands-on experience and structured clinical training.

Most respondents (63.9%) reported no formal trauma-management training after graduation, and over half had not attended continuing education courses. These findings align with the broader observation that lack of experience is the most frequently cited factor contributing to low confidence in both primary and permanent dentitions.

Encouragingly, most participants expressed interest in additional training, with preferences for online courses, hands-on workshops, and face-to-face lectures. Prior studies have demonstrated that targeted educational programs can significantly enhance TDI-related knowledge and confidence (34). A regional survey also identified significant deficiencies in undergraduate trauma-management curricula across the Arabian Peninsula (35). Importantly, the Australian study by Jadav and Abbott (26) found that familiarity with the IADT guidelines was the only factor significantly associated with both the number of trauma cases treated and self-reported knowledge, highlighting the critical role of guideline awareness.

In the UAE, awareness of the IADT guidelines and DTG was moderate (50.5%), indicating significant room for improvement. Greater promotion of these evidence-based tools, particularly in multiple languages, may support better integration into routine clinical practice. Ensuring easy access to guideline content and facilitating its use in decision-making are essential steps toward improving trauma-care quality.

Regarding guideline utilization, practitioners most frequently consulted the treatment recommendation and follow-up protocol sections of the IADT guidelines, while prognosis guidance was underused despite its relevance to long-term management planning.

Responses to the case scenarios revealed important knowledge gaps. In the complicated crown-fracture scenario, a notable proportion selected inappropriate treatments, such as pulp extirpation or a zinc oxide-eugenol pulpotomy medicament. Similarly, almost one-third recommended unnecessary extraction for intruded primary incisors, demonstrating limited awareness of the 2020 IADT update. These trends parallel those reported by Elwerfelli et al. (12), who found similar patterns across GCC countries. Holan and Needleman (36) emphasized that unnecessary extraction of primary incisors can lead to avoidable complications and negatively affect the child's quality of life.

Strengths of the study

As with all survey-based studies, the following limitations should be acknowledged. Data collected through electronic surveys may be subject to response bias and recall inaccuracies. The non-random sample and overrepresentation of female dentists may have influenced some comparisons. Furthermore, the predominance of respondents from Dubai may limit the generalizability of findings across the UAE.

## Conclusion

This study underscores significant deficiencies in both confidence and knowledge among dental practitioners in the UAE regarding the management of traumatic dental injuries (TDIs). The frequent use of outdated materials and deviations from current IADT guidelines in clinical scenarios raise concerns about compromised patient care. These findings underscore the pressing need for structured educational initiatives and the broader dissemination of updated, evidence-based resources to support informed clinical decision-making and enhance treatment outcomes.

## Data Availability

The raw data supporting the conclusions of this article will be made available by the authors, without undue reservation.

## Supplementary material

Supplementary Table S1 Questionnaire used in the study.
