# Supplementary material for: The effect of dental trauma management resources on dental practitioners’ self-reported confidence and knowledge in the United Arab Emirates
Source: Front Dent Med. 2026 Jul 16;7:1883091. doi: 10.3389/fdmed.2026.1883091 (PMC13422386; doi:10.3389/fdmed.2026.1883091)
Supplement: Supplementary Table S1 — Questionnaire used in the study. [file Table1.docx]

**Supplementary Table S1. Structure and Detailed Items of the Questionnaire**

| Section | Domain | Question / Item | Response Format |
| --- | --- | --- | --- |
| Section 1 | Demographics | Age | 20–30 / 31–40 / 41–50 / >50 |
|  | Demographics | Gender | Male / Female |
|  | Demographics | Emirate of practice | Abu Dhabi / Dubai / Sharjah / Ajman / RAK / Fujairah / UAQ |
|  | Professional background | Specialty | GDP / Pediatric Dentist / Endodontist / Other |
|  | Professional background | Type of practice | Government / Private / Academic / Other |
|  | Professional background | Years of experience | ≤5 / 6–10 / 11–20 / ≥21 |
|  | Professional background | Working hours per week | ≤7 / 8–14 / 15–21 / 22–28 / 29–35 |
| Section 1 | Exposure | Average number of trauma cases in 3 months | 0–4 / 5–12 / 13–24 / >24 |
|  | Training | Postgraduate trauma training | Yes / No |
|  | Training | Attendance of CE courses | Yes / No |
|  | Training | Access to pediatric dentist | Yes / No |
|  | Awareness | Awareness of IADT 2020 guidelines | Aware & read / Aware not read / Unaware |
|  | Awareness | Awareness of DTG | Yes / No |
|  | Utilization | Frequency of DTG use | Rarely / Sometimes / Often / Almost always |
|  | Confidence (Primary) | Uncomplicated crown fracture | Likert 1–5 |
|  | Confidence (Primary) | Complicated crown fracture | Likert 1–5 |
|  | Confidence (Primary) | Luxation injuries | Likert 1–5 |
|  | Confidence (Primary) | Avulsion | Likert 1–5 |
|  | Confidence (Permanent) | Partial pulpotomy (Cvek) | Likert 1–5 |
|  | Confidence (Permanent) | Repositioning intruded teeth | Likert 1–5 |
|  | Confidence (Permanent) | Splinting | Likert 1–5 |
|  | Confidence (Permanent) | Regenerative procedures | Likert 1–5 |
|  | Confidence (Permanent) | MTA apical plug | Likert 1–5 |
| Section 2 | Scenario 1 | Complicated crown fracture management | Multiple choice |
| Section 2 | Scenario 2 | Intruded primary incisor management | Multiple choice |
| Final | Education | Interest in further training | Yes / No |
|  | Education | Preferred learning method | Online / Workshop / Lecture / None |
